# Rare Occurrence of Inhibitors in Von Willebrand Disease: A Case Report

**DOI:** 10.3389/fmed.2021.807664

**Published:** 2022-01-11

**Authors:** Bipin P. Kulkarni, Kirti Ghargi, Chandrakala Shanmukhaiah, Shrimati D. Shetty

**Affiliations:** ^1^ICMR- National Institute of Immunohaematology, KEM Hospital Campus, Mumbai, India; ^2^Department of Haematology, KEM Hospital, Mumbai, India

**Keywords:** VWD, inhibitors, quantitation, Bethesda assay, rare occurrence

## Abstract

**Introduction:** Type 3 Von Willebrand Disease (VWD) is the least common but the most severe form of a disease, with a prevalence of about 0. 5 to 1 per million in Western countries. The prevalence of type 3 VWD in the developing countries, with a high degree of consanguinity, is about 6 per million. Moreover, due to underdiagnosis of the milder cases, the prevalence of type 3 VWD is about 50% of the cases. Rarely, some patients develop the Von Willebrand Factor (VWF) inhibitors, which may subsequently develop severe anaphylactic reactions on further exposure to the VWF containing factor replacement therapy. The prevalence of inhibitor development in patients with type 3 VWD has been shown to be in the range of 5.8 to 9.5%. In the absence of a gold standard assay for the quantitation of VWF inhibitors, a correct diagnosis and management of these patients are often challenging.

**Objectives:** The objective of this study is to standardize the Bethesda assay for the VWF inhibitors and to estimate the VWD inhibitor titer in two cases of congenital type 3 VWD, which developed the VWF inhibitors.

**Results and Conclusions:** We could successfully standardize the Bethesda assay for the quantitation of VWF inhibitors in two patients with congenital type 3 VWD with inhibitors.

## Case 1

A 5-year-old female child has a known case of congenital type 3 VWD, born of consanguineous marriage, and with a homozygous deletion of whole VWF gene, as shown by NGS- exome sequencing. She presented with a history of fall, hurting the left side of her jaw, and developed a painful swelling of the left jaw with gradual extension, resulting in severe pain. On clinical examination, she showed swelling, which was tender and warm, extending from the body of mandible to an angle measuring approximately 3 x 4 cm. She was treated with Intermediate Purity Factor (IPF) VIII concentrate (Immunate, 2:1 ratio of FVIII and VWF; Koate, 1:1 ratio of FVIII and VWF) at 20 IU/kg twice weekly for 2 weeks. However, the swelling and pain started worsening, as well as the intermittent gum bleeding. The duration of infusion was increased to thrice a week. The patient did not show progress and she continued to have gum bleeding. She was posted for curettage of the pseudo tumor. She developed itching and has rashes all over body. Due to the sub-optimal response to treatment and to allergic reaction, the presence of the VWF inhibitors was suspected.

## Case 2

A 25-year-old woman phenotypically diagnosed at the age of 5 years old, has a congenital type 3 VWD, was born to a 2nd degree consanguineous parents, presented with recurrent episodes of ecchymosis, heavy menorrhagia, and melena. She had multiple episodes of severe melena and was presented with shock. On infusion of IPF (Immunate, 2:1 ratio of FVIII and VWF) at 20 IU/kg twice weekly for 2 weeks, there was no response. She had 50 days of exposure to the IPF. She was referred to this center with the suspected VWF inhibitors.

## Laboratory Investigations

### Screening for VWF Inhibitors

The baseline screening coagulation assays, APTT mixing, Von Willebrand Factor: Antigen (VWF:Ag), Von Willebrand Factor: Ristocetin Cofactor (VWF:RCo), and Factor VIII: Clotting (FVIII:C) assays were performed on the plasma samples of the patients, alongside normal pooled plasma (NPP) (Table 1). Another known inhibitor, the patients with the negative type 3 VWD plasma with VWF: Ag level of 1.8%, was tested alongside as test control. The plasma of the patients and the test control plasma were separately mixed with an NPP, in a 1:1 dilution (150 μl pt./test control plasma + 150 μl of NPP) and incubated at 37°C for 1 h. After incubation, the VWF: Ag levels and the VWF:RCo activity were assayed on an ACL TOP 300 automated coagulation analyzer (Instrumentation Laboratory, Lexington, MA, USA) (Table 2).

**Table 1:** Baseline screening coagulation, APTT mixing, VWF:Ag, VWF:RCo, and FVIII:C assays performed on NPP and on plasma samples of the patients.

**Parameters**	**Normal pooled plasma (NPP) control**	**Case 1**	**Case 2**
PT	13.7 s	15.5 s	14.4 s
APTT	26.6 s	47 s	45 s
APTT mixing	-	38.7 s	30.8 s
VWF: Ag	113.10%	<2.2%	<2.2%
VWF:RCo	111.40%	1.60%	2.40%
Factor VIII	152%	1.80%	2.60%

**Table 2:** Mixing studies.

	**Test control + NPP**	**Case 1 + NPP**	**Case 2 + NPP**
VWF:Ag	54.8%	<2.2%	24.6%
VWF:RCo	52.4%	0.0%	0.0%

### VWF Inhibitor Assay

There is no gold standard assay for the identification and quantification of VWF inhibitors. Hence, the approach taken was based on the principle of combining the studies, followed by the Bethesda assay, to estimate the VWF inhibitors (1). The inhibitor titer was then derived from the Bethesda graph, based on the residual VWF:RCo activity.

The plasma samples of the patients were serially diluted using the Owren Koller (OK) buffer. Each of the dilution was mixed with an equal volume of NPP. A Buffer Blank, containing NPP and OK buffer, was taken as a reference control. The serially diluted plasma of the patients and the reference control reactions were incubated at 37°C for 2 h. After incubation, the reference control and the serial dilutions of the patients' plasma were diluted to obtain 1:5 and 1:10 dilutions with OK buffer. The VWF:RCo activity in control dilutions was determined using a ACL-VWF:RCo kit (Instrumentation Laboratory Company - Lexington, MA 02421-3125, USA), and was compared with each of the serial dilutions of the patients' plasma (Table 3).

**Table 3:** Residual VWF:RCo as determined by the nearest dilution factor of 1:5 dilution of the patient, to the 1:10 dilution of the reference control.

	**Case 1**		**Case 2**	
	**VWF:RCo (%)**		**VWF:RCo (%)**	
Assay dilutions	1:5	1:10	1:5	1:10
OK + NPP (Reference control)	17.5	8.0	18.3	8.7
Patient plasma 1:2 dilution	0.0	-	0.0	
Patient plasma 1:4 dilution	0.0	-	7.5	3.0
Patient plasma 1:8 dilution	0.0	-	14.2	6.7
Patient plasma 1:64 dilution	0.0	-	-	-
Patient plasma 1:128 dilution	5.4	2.4	-	-
Patient plasma 1:256 dilution	13.0	6.1	-	-

The detailed protocol is as given below:

1. The plasma samples of the patients were incubated at 56°C for 30 min to eliminate any residual VWF by heat inactivation, whereas the antibodies are heat resistant. The plasma of the patients were then centrifuged at 4,000 rpm for 15 min and the supernatant plasma, containing the VWF inhibitors, was harvested for testing, leaving out the heat-coagulated pellet of the plasma proteins.
2. The plasma of the patients obtained through step 1 was serially diluted with OK buffer to dilutions of 1:2, 1:4, and up to 1:256, to serially dilute the VWF inhibitors that were present in the patients' plasma.
3. Each dilution was mixed with NPP in 1:1 proportion in siliconized glass tubes.
4. The NPP, with OK buffer in 1:1 proportion, was taken as a reference control.
5. All tubes in points 3 and 4 above were incubated at 37°C for 2 h.
6. After incubation, 1:5 and 1:10 dilutions were made from each tube with OK buffer.
7. The VWF:RCo activity was determined in each dilution on ACL TOP 300 automated coagulation analyzer (Instrumentation Laboratory, Lexington, MA, USA), and the results were recorded and analyzed as explained in the section below.

Table 2 shows 50% correction in the test control VWF:Ag and VWF:RCo levels, suggesting the absence of VWF inhibitors in the test control plasma of the patients. However, the patients with possible VWF inhibitors showed no correction in VWF:RCo levels. However, in case 2, the VWF:Ag was not completely inhibited. This indicated the presence of VWF inhibitors in the plasma of the patients, more so against the VWF:RCo activity, possibly because of the effect of inhibitors on large multimers. Both the patients were negative for FVIII:C inhibitors.

From Table 3, it was observed that in Case 1, the VWF inhibitor titer was between the residual VWF:RCo % activity corresponding to the dilution factors of 1:128 and 1:256; while in Case 2, the inhibitor titer was between the dilution factors of 1:4 and 1:8.

### The Bethesda Assay

One Bethesda unit is defined as the concentration of inhibitors that neutralize 50% of the factors in NPP/reference plasma.

In the FVIII inhibitor Bethesda assay, which is a clot-based assay, the clotting time of the patients' plasma decreases as we go higher with inhibitor dilutions, to a point where the 1:5 dilution clotting time of the patients is the closest to the 1:10 dilution clotting time of the reference controls. The residual FVIII:C value is then obtained from the factor assay graph and is plotted on the Bethesda graph to obtain the corresponding Bu/ml, which is multiplied with the dilution factor to obtain the final Bu/ml titer.

**Figure 1:**
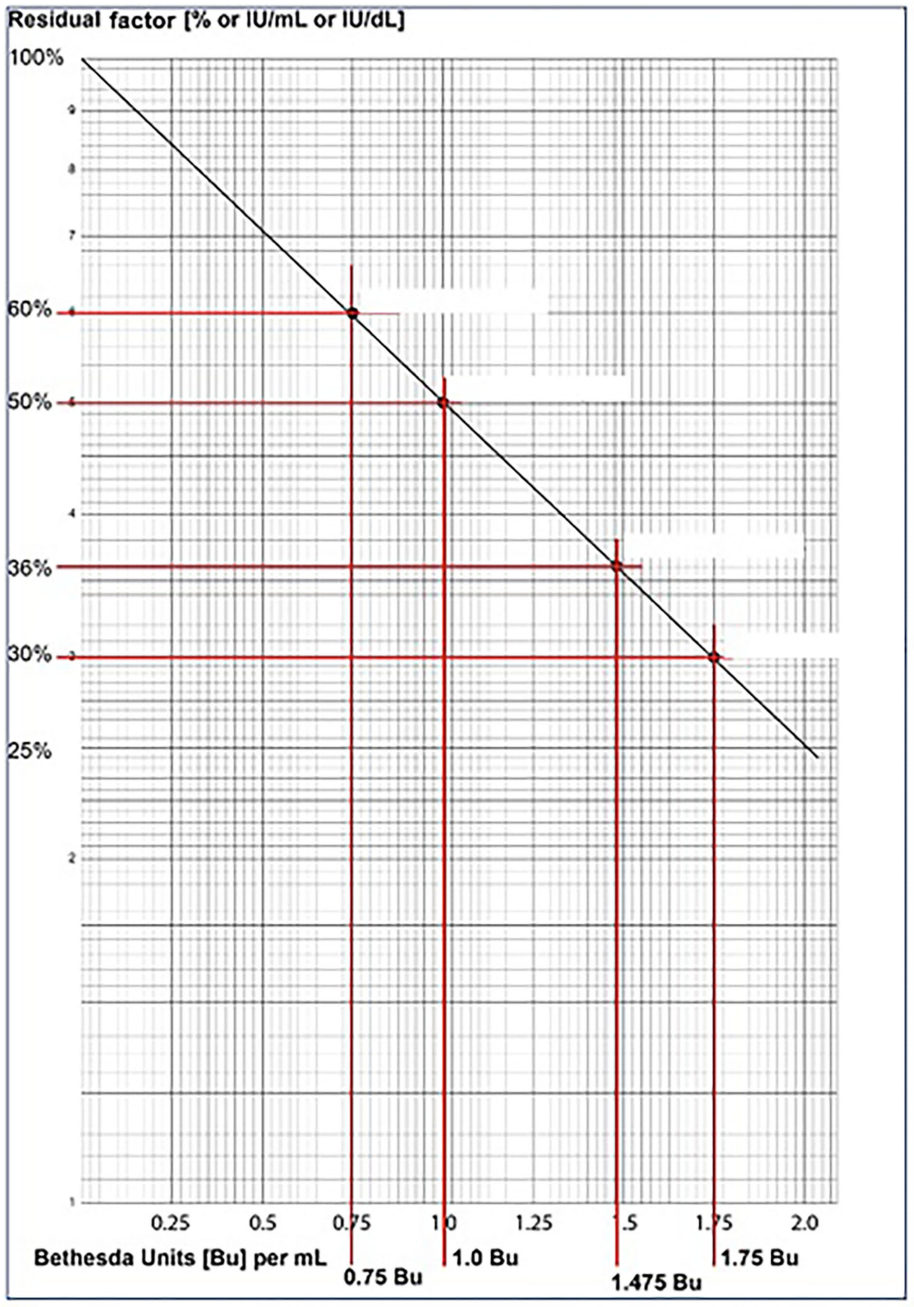
Shows a representative Bethesda assay graph for plotting the calculated residual VWF:RCo % activity, to derive the inhibitor titer in Bu/ml, which is then multiplied with the dilution factor to obtain the final VWF inhibitor titer in Bu/ml.

As per Bethesda assay principle, the 1:10 dilution corresponds to 50% VWF:RCo activity value. In Table 3, Case 1: Reference Control, the 1:10 dilution value of 8% corresponds to 50%. Therefore, the patient's value of 5.4% corresponds to 50 x 5.4 / 8 = 33.75% (residual VWF:RCo activity), and has obtained the corresponding Bu/ml value of 1.7 from the graph (Figure 1). The 1.7 x dilution factor of 128 = 217.6 Bu/ml was the VWF:RCo inhibitor Bethesda level of the Case 1.

Similarly, in Case 2: Reference Control, the 1:10 dilution value of 8.7% corresponds to 50%. Therefore, the patient's value of 7.5% corresponds to 50 x 7.5/8.7 = 43.10% (residual VWF:RCo activity), and has obtained the corresponding Bu/ml value of 1.2 from the graph (Figure 1). The1.2 x dilution factor of 4 = 4.8 Bu/ml was the VWF:RCo inhibitor Bethesda level of the Case 2.

## Discussion

Development of inhibitors to a VWF in a congenital type 3 VWD patient, followed by either recombinant or intermediate purity of the VWF containing Factor VIII concentrate, is a rare incidence. Due to underdiagnosis of the milder cases, the prevalence of type 3 VWD is about 50% of the cases (2–4). Rarely, some patients develop the Von Willebrand Factor (VWF) inhibitors, which may subsequently develop severe anaphylactic reactions on further exposure to the VWF containing factor replacement therapy (5, 6). The prevalence of inhibitor development in patients with type 3 VWD has been shown to be in the range of 5.8 to 9.5% (7–12).

The first case of a severe type 3 VWD with inhibitors was reported in 1974 (13). Subsequently, more studies on type 3 VWD with inhibitors were reported (14). A recently large study on 99 Iranian patients with type 3 VWD reported 19.2% positivity for VWF inhibitors, which is substantially higher than the earlier reports (15). Most of the reports of VWF inhibitors were from patients with partial or complete VWF gene deletions. However, subsequent publications have reported patients with non-sense, frameshift mutations, and partial or complete gene deletions. Moreover, not all cases of partial gene deletions develop alloantibodies against VWF, which was described in a study of 25 patients with type 3 VWD cases, of which, five had homozygous partial large deletions, and yet did not develop VWF inhibitors as a complication to treatment (1).

Once a patient develops the inhibitor, any further exposure to VWF can give rise to severe anaphylactic reactions (1). Moreover, there is no standardized assay available for the reliable detection and quantitation of the VWF inhibitors. This can potentially cause delays before the laboratory diagnosis of the VWD inhibitors is made, jeopardizing the treatment of the patients who were bleeding and posing a life-threatening situation. The available assays are based on the principle of combined studies to demonstrate the inhibition of the platelet-dependent function of VWF. It has been recommended to broadly evaluate the VWF function. Some laboratories have used an enzyme-linked immunosorbent assay (ELISA). These assays appear to be sensitive, however, there is a concern about their high rate of false positivity (1, 16–18). The mixing studies with NPP as control, followed by a Bethesda assay, correlated well with the inhibitor titres, as reflected in the clinical outcomes of the patients. After the VWF inhibitor assays, the Case 1 has underwent a surgery for her pseudotumor and was given rFVIIa (90 mcg/kg body weight) just prior to surgery. After 30 min of starting the surgery, she had an uncontrolled bleeding from the wound and again was started on of rFVIIa (90 mcg/kg body weight). On day 3, she had a persistent swelling of the jaw and her platelet count had dropped to 10 x 10^9^/L. In view of the severe thrombocytopenia, IVIG (1 g/kg body weight) was given, along with methylprednisolone. Platelet count increased to 75 x 10^9^/L. As her response to rFVIIa was unsatisfactory, she was treated with rituximab 375 mg/m^2^ weekly, once a week for 4 weeks. Though there was reduction in bleeding, she still continued to have swelling on her jaw. She was then started on emicizumab (3 mg/kg body weight) once a week for 1 month followed by once in 2 weeks. She was given 4 doses of emicizumab, after which, her swelling subsided completely. She completed 16 weeks of treatment and she did not have any breakthrough bleeding, neither required for blood nor Clotting Factor Concentrate (CFC) support.

The second patient was treated with rFVIIa + rFVIII. Both the patients responded well to the treatment and recovered.

Some of the treatment modalities successfully used are rFVIII, rFVIIa, rFVIIa, and rFVIII, Platelet infusions, APCC, Antifibrinolytics, and even Immune Tolerance Induction (ITI) have been successfully attempted with VWF/F8 Conc. and IVIG with Corticosteroids (1).

The effect of alloantibodies on the functionality of VWF needs to be studied, as the antibodies may have different epitope specificities on the VWF molecule. A regular inhibitor screening in patients with type 3 VWD on requiring frequent infusions may be considered.

The limitation of this assay is that we could not compare and validate the VWF inhibitor titer with a gold standard assay. Also, a negative result does not rule out VWF inhibitors. The inhibitors could be directed to other functional or non- functional domains of the VWF. Hence, VWF: Collagen Binding (VWF:CB) assay is also recommended (1). However, the standardized assay is robust, reproducible, and the inhibitor titer correlates well with the clinical manifestations of the patients.

## Conclusion

Inhibitors to VWF in type 3 VWD patients is not a common occurrence. We have not only identified 2 such patients of type 3 VWD who developed inhibitors, but also could reliably estimate the inhibitor titer by adapting the Bethesda assay for VWF inhibitors, which we have described in detail.

## Data Availability Statement

The original contributions presented in the study are included in the article/supplementary material, further inquiries can be directed to the corresponding author/s.

## Ethics Statement

The studies involving human participants were reviewed and approved by ICMR- NIIH IEC, ICMR- National Institute of Immunohaematology. Written informed consent to participate in this study was provided by the participants' legal guardian/next of kin.

## Author Contributions

BK analyzed the data and wrote the manuscript. KG performed the laboratory assays. CS recruited and treated the patients. SS gave expert inputs and analyzed the data. All authors contributed to the article and approved the submitted version.

## Conflict of Interest

The authors declare that the research was conducted in the absence of any commercial or financial relationships that could be construed as a potential conflict of interest.

## Publisher's Note

All claims expressed in this article are solely those of the authors and do not necessarily represent those of their affiliated organizations, or those of the publisher, the editors and the reviewers. Any product that may be evaluated in this article, or claim that may be made by its manufacturer, is not guaranteed or endorsed by the publisher.
